# A Universal Formulary, Containing the Methods of Preparing and Administering Officinal and Other Medicines; the Whole Adapted to Physicians and Pharmaceutists

**Published:** 1850-04

**Authors:** 


### BIBLIOGRAPHICAL NOTICES.

---

*A Universal Formulary, containing the methods of preparing and administering officinal and other medicines; the whole adapted to physicians and pharmaceutists.* By R. EGLESFELD GRIFFITH, M.D. Selecta sunt quæ medicum nobilitant. Linnæus. 8vo. pp. 567. Philadelphia, Lea & Blanchard, 1850.

